# Long COVID: An approach to clinical assessment and management in primary care

**DOI:** 10.4102/safp.v65i1.5751

**Published:** 2023-06-23

**Authors:** Rubeshan Perumal, Letitia Shunmugam, Kogieleum Naidoo

**Affiliations:** 1 Centre for the AIDS Programme of Research in South Africa, Faculty of Medicine, University of KwaZulu-Natal, Durban, South Africa; 2 Department of Pulmonology, Faculty of Medicine, University of KwaZulu-Natal, Durban, South Africa

**Keywords:** Long COVID, long-haul COVID, post-acute sequelae of SARS-CoV-2, COVID-19, SARS-CoV-2

## Abstract

Long COVID is an emerging public health threat, following swiftly behind the surges of acute infection over the course of the COVID-19 pandemic. It is estimated that there are already approximately 100 million people suffering from Long COVID globally, 0.5 million of whom are South African, and for whom our incomplete understanding of the condition has forestalled appropriate diagnosis and clinical care. There are several leading postulates for the complex, multi-mechanistic pathogenesis of Long COVID. Patients with Long COVID may present with a diversity of clinical phenotypes, often with significant overlap, which may exhibit temporal heterogeneity and evolution. Post-acute care follow-up, targeted screening, diagnosis, a broad initial assessment and more directed subsequent assessments are necessary at the primary care level. Symptomatic treatment, self-management and rehabilitation are the mainstays of clinical care for Long COVID. However, evidence-based pharmacological interventions for the prevention and treatment of Long COVID are beginning to emerge. This article presents a rational approach for assessing and managing patients with Long COVID in the primary care setting.

## Introduction

By the last quarter of 2022, over 700 million people had been infected by severe acute respiratory syndrome coronavirus 2 (SARS-CoV-2) in a global pandemic that claimed the lives of over six million people. For many coronavirus disease 2019 (COVID-19) survivors, recovery from the acute illness has been incomplete or temporary.^1^ Persistence of initial symptoms, recurrence of previously resolved symptoms or the emergence of new symptoms has characterised the post-COVID-19 journey of millions of people worldwide. The development of Long COVID may follow asymptomatic, mild, moderate, severe or critical acute COVID-19.^2,3^ Although there is a gradient of risk for developing Long COVID proportional to severity of the acute illness, the sheer volume of individuals who suffered asymptomatic or mild acute COVID-19 has resulted in the majority of Long COVID sufferers emerging from these groups. Severe acute respiratory syndrome coronavirus 2 endemicity appears likely, and future disease transmission will depend on the interplay between virus evolution, host immunity and the population’s susceptibility to COVID-19. Even if we are able to avert future resurgences with highly pathogenic variants of SARS-CoV-2, the risk of Long COVID from asymptomatic and mild acute COVID-19 remains significant.^4^

### Recovery from acute COVID-19

Recovery from acute COVID-19 has been variable, depending on SARS-CoV-2 variant, host factors, vaccination status, and the number of prior infections with SARS-CoV-2. We estimate that there are already approximately 100 million people suffering from Long COVID globally, 0.5 million of whom are South African, and for whom our limited understanding of the condition has forestalled appropriate diagnosis and clinical care.^1,5,6,7^ Initial estimates, early in the pandemic, suggested that up to 75% of critically ill patients, half of all hospitalised patients, and up to 30% of patients with mild or asymptomatic disease may develop Long COVID.^4,6,8,9,10,11,12^ More recent estimates, reflecting greater vaccination coverage and the predominance of the Omicron lineages, suggest a prevalence of approximately 5% – 10% following SARS-CoV-2 infection, incorporating all levels of acute illness severity. In South Africa, 46.7% of hospitalised and 18.5% of non-hospitalised patients developed Long COVID across the Beta, Delta and Omicron waves.^6^ Similar to global cohorts, the proportion of South African patients who developed Long COVID was significantly lower following the Omicron wave than the Delta or Beta waves (18.5% vs ~60%).^6^ The leading risk factors for developing Long COVID include severe acute COVID-19, re-infection with SARS-CoV-2, female gender, advancing age, atopy, multimorbidity, obesity, diabetes mellitus, HIV and other causes of immunosuppression. Importantly, however, Long COVID may occur with all acute phase disease severities, and therefore the greatest burden of disease in absolute numbers is among middle-aged patients with no pre-existing medical conditions, most of whom suffered mild or moderate acute COVID-19 for which hospitalisation was not necessary.^13,14^ The temporal evolution of the diverse clinical phenotypes associated with Long COVID exhibits significant heterogeneity. The majority of symptoms recur after resolution of the acute illness or increase in intensity from around 1 month after the acute infection. Neurological and cognitive symptoms (including brain fog, memory impairment, depression and anxiety) may increase in prevalence or intensity over time, respiratory symptoms remain stable or decline slowly over time, while gastrointestinal symptoms are more likely to resolve completely by 12 months.^15^ Many other suspected Long COVID phenotypes, such as rheumatological (joint pain, back pain and thigh pain), dermatological (hair loss and skin rash) and ophthalmological (blurred vision), may have a remote onset in relation to the acute illness, occurring several months after an initial abatement of the acute illness. Over the longer term, less than 15% of patients with Long COVID report complete recovery at 1 year after symptom onset, and for whom the prognosis remains uncertain.^16^ For some Long COVID phenotypes, including postural orthostatic tachycardia syndrome, mast cell activation syndrome and chronic fatigue syndrome, the condition may likely be lifelong.

### Defining Long COVID

Although there are several case definitions for Long COVID, no universally accepted definition for Long COVID exists. The South African Ministerial Advisory Committee on COVID-19 supported the use of the World Health Organization (WHO) Consensus Clinical Case Definition, which states that Long COVID represents:

[A*] broad range of symptoms (including physical and mental manifestations) and symptom clusters that persist or develop at least three* months from the onset of COVID-19, last for more than 2 months, have an impact on the patient’s everyday functioning, and are not explained by an alternative diagnosis.^17,18^

A diversity of clinical phenotypes has been recognised, ranging from symptom clusters, discrete clinical events, organ-specific presentations and multi-system syndromes.^19,20,21,22,23,24^ The collection of symptoms and clinical problems experienced by patients likely signifies a new syndrome unique to the SARS-CoV-2 virus; however, overlapping of clinical phenotypes have been observed with well-described conditions, namely post-intensive care unit (ICU) syndrome, myalgic encephalomyelitis or chronic fatigue syndrome (ME/CFS) and postural orthostatic tachycardia syndrome (Table 1)^18,25,26,27^.

**TABLE 1:** An overview of conditions that overlap with the symptom profile of Long COVID.

Condition	Clinical diagnostic guide
Myalgic encephalomyelitis or chronic fatigue syndrome (ME/CFS)	All of the following: Extreme fatigue Disrupted sleep pattern Post-exertional malaise *and* at least one of the following: Cognitive impairment including poor concentration, brain fog, memory impairment Orthostatic intolerance
Post-intensive care syndrome (PICS)	Cognitive sequelae: memory impairment, brain fog, speech difficulty and poor concentration. Mental health sequelae: post-traumatic stress, anxiety, insomnia and depression. Physical sequelae: muscle weakness, fatigue, decreased mobility and shortness of breath.
Postural orthostatic tachycardia syndrome or inappropriate sinus tachycardia (POTS/IST)	Light-headedness, exercise intolerance, fatigue, headache, sweating, tremor, anxiety and palpitations. Elevated heart rate (> 30 beats per minute, within 5–10 min of standing or upright tilt, without orthostatic hypotension)

*Source:* Please see the full reference list of the article Sisó-Almirall A, Brito-Zerón P, Ferrín LC, et al. Long covid-19: Proposed primary care clinical guidelines for diagnosis and disease management. Int J Environ Res Public Health. 2021;18(8):4350. https://doi.org/10.3390/ijerph18084350, for more information

### Potential biological mechanisms

The pathogenesis of Long COVID remains incompletely elucidated. However, several potential biological mechanisms have been proposed to underpin the development of Long COVID. These include persistence of replication competent virus, persistence of virus macromolecules and proteins, platelet microclot formation with microcirculatory insufficiency, autoimmunity directed at both nuclear and extracellular protein targets, dysbiosis, mast cell activation syndrome, reactivation of latent herpesviruses, persistent metabolic derangement and persistent central nervous system inflammation.

*Viral persistence* has been demonstrated in individuals recovering from COVID-19 weeks, and sometimes months, after the acute infection. Severe acute respiratory syndrome coronavirus 2 ribonucleic acid (RNA), including subgenomic RNA (a marker of recent viral replication), has been detected in urine, blood, upper respiratory tract and stool, in patients with persistent symptoms up to 4, 8, 12 and 18 weeks, respectively, following the acute illness. An autopsy study revealed the long-term persistence of SARS-CoV-2 RNA across a wide range of human tissues, most abundantly in adrenal glands, kidneys, intestine, lymph nodes, spleen and heart. Severe acute respiratory syndrome coronavirus 2 antigens have the potential to act as superantigens resulting in overstimulation of the immune system with consequent chronic inflammation and periodic cytokine hypersecretion, ineffective viral clearance and recurrent immune exhaustion.^28^ Viral cytopathy and inflammation, depending on their extent, may give rise to organ-specific and systemic symptoms.

The SARS-CoV-2 may directly and indirectly interact with platelets to produce amyloidogenic platelet aggregates.^29^ Microclots, comprising anomalous platelet aggregates that are highly resistant to fibrinolysis, have been demonstrated in peripheral blood from patients with Long COVID. In addition, these microclots leach pro-inflammatory cytokines contributing to a persistent state of hypercoagulability and hyperinflammation. Dissemination, deposition and accumulation of microclots within the various microcirculations may explain the diverse multi-system clinical phenotypes associated with Long COVID.^30^

Severe acute respiratory syndrome coronavirus 2 triggers *mast cell activation* and degranulation resulting in the release of vasoactive and pro-inflammatory mediators including growth factors, chemokines, cytokines, heparin and histamine, which contribute to the hyperinflammatory milieu characteristic of acute COVID-19. Persistent pulmonary mast cell activation may occur beyond the acute COVID-19 phase, even after the attainment of polymerase chain reaction (PCR)-negativity for SARS-CoV-2. Lung and myocardial post-mortem studies of COVID-19 patients demonstrated a significantly elevated quantity of mast cells in these organs during infection.^31,32^ Patients with Long COVID develop similar symptoms to patients with Mast Cell Activation Syndrome and demonstrate improvement with histamine receptor antagonists.^33,34^

Serum obtained from patients with Long COVID reveals elevated levels of *autoantibodies* to extractible nuclear antigens up to 3 and 6 months after acute COVID-19. Persistent antibodies against U1-snRNP and SS-B/La, for example, substantially correlate with proinflammatory cytokines, biomarkers of inflammation and D-dimer levels in these patients. Long COVID may therefore represent a virus-induced autoimmune process in which autoantibodies act as intermediate pathogenic factors producing the diverse clinical phenotypes associated with Long COVID.^35^

Severe acute respiratory syndrome coronavirus 2 infection directly disrupts the gut microbiome causing *dysbiosis* and secondary bacterial infections.^36^ Dysbiosis has been demonstrated in stool samples from Long COVID patients, revealing significantly diminished levels of beneficial gut microbiome species, causing an imbalanced gut microbe environment at 6 months post-acute COVID-19 infection and associated with increased levels of systemic inflammation. Dysbiosis, accompanied with heightened levels of inflammation, is detectable beyond 12 months after the acute infection, and is strongly associated with persistent fatigue, memory impairment and insomnia.^37^

Herpes viruses such as Epstein Barr Virus (EBV) and Human Herpesvirus-6 (HHV-6) are the most common immunotropic viruses that utilise latency as a mechanism to evade the host’s immune system. Serological samples from Long COVID patients revealed the presence of herpesvirus DNA: 42.6% EBV, 25% HHV-6 and 32.4% EBV + HHV-6.^38^ *Reactivation of herpesviruses* leads to systemic inflammation, immunological dysfunction, and increased virus replication, which may give rise to the distinct clinical phenotypes expressed in Long COVID.^39^

Emerging evidence suggests that *metabolic dysfunction* coupled with chronic inflammation may potentiate the onset of Long COVID.^40^ Blood plasma isolated from Long COVID patients demonstrated dysregulation of several cytokines, lipoproteins and metabolites. Overall, energy metabolism dysfunction and dysregulation of inflammatory processes could potentially contribute to the emergence of Long COVID.^41^

*Neuroinflammation*, induced through systemic inflammation, gives rise to glial dysregulation and neural function impairment. Systemic inflammatory mediators directly affect the cellular plasticity of the brain by activating microglia, inducing the generation of neurotoxic astrocyte reactivity, and prompting oligodendrocyte and neuronal cell death. High levels of circulating CCL11 may contribute to cognitive impairment through restriction of neurogenesis in the hippocampus, inhibition of myelinating oligodendrocytes leading to myelin loss, and interruption of neural circuit signalling.^42,43^

### Common clinical phenotypes

**FIGURE 1:**
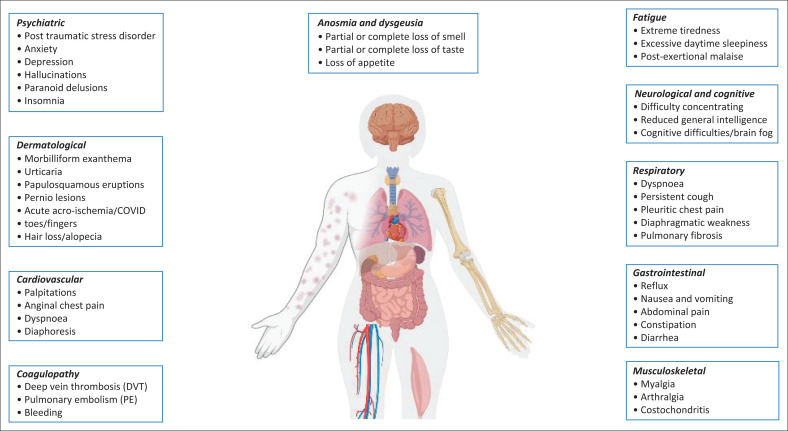
Clinical sequalae associated with Long COVID.

Post-acute sequelae and an increased burden of new medical diagnoses have been reported in up to 80% of patients following infection with SARS-CoV-2^44^ (Figure 1). Extreme fatigue or post-exertional malaise represents the most reported persistent complaints across global Long COVID cohorts, occurring in 60% – 70% of affected individuals.^45^ Persistent dyspnoea and cough may occur in up to 60% of patients and may persist with gradual improvement over time. The global prevalence of gustatory and olfactory dysfunction during acute COVID-19 infection is estimated to be 50%. Most patients recover considerably quickly; however, a few patients report little to no improvement during the 1st months post-acute COVID infection, with the prevalence of persistent anosmia and dysgeusia reported to be approximately 20% – 30%, 8 months after acute infection.^46^ Persistent cardiovascular symptoms occur in 10% of patients previously hospitalised with acute COVID-19. This clinical phenotype is associated with an increased risk of major adverse cardiovascular events and arrhythmias up to 12 months after acute infection. Neuropsychiatric symptoms of Long COVID can occur after asymptomatic, mild, moderate or severe acute COVID-19, ranging in prevalence between 40% and 70%, with about one-third of patients reporting ongoing neurological or psychiatric symptoms 6 months after acute COVID-19. Cognitive impairment has been consistently demonstrated in over 50% of patients with Long COVID.^47^ Musculoskeletal and rheumatic symptoms occur in approximately 40% of Long COVID patients at 6 months after acute infection.^48^ Most skin rashes associated with COVID-19 emerge within the inflammatory phase of the initial infection, between days 7 and 14. Some dermatological manifestation such as COVID-toes and urticaria, may persist for months.^49^ The prevalence of Long COVID-related gastrointestinal symptoms ranges between 10% and 80%, with abdominal pain and diarrhoea predominating.^50^

### Increased risk of new medical conditions and discrete clinical events

Patients who recover from acute COVID-19 remain at heightened risk for new medical conditions and discrete, potentially life-threatening clinical events.^51,52,53,54^ Table 2^51,52,53,54^ lists the discrete clinical events or new major medical diagnoses that occur with significantly increased incidence in individuals following COVID-19 compared with risk-matched historical or contemporary controls with no documented prior infection with SARS-CoV-2.

**TABLE 2:** Discrete clinical events and major medical diagnoses occurring with increased incidence following infection with SARS-CoV-2.

Disorder category	Specific diagnoses
Ischaemic heart disease	Acute coronary syndrome, ischaemic cardiomyopathy, angina
Dysrhythmias	Atrial fibrillation, sinus tachycardia, sinus bradycardia, ventricular arrhythmias, atrial flutter
Inflammatory disease of the heart/pericardium	Pericarditis, myocarditis
Other cardiovascular disorders	Heart failure, non-ischaemic cardiomyopathy, cardiac arrest, cardiogenic shock
Cerebrovascular disorders	Stroke, transient ischaemic attack (TIA), haemorrhagic stroke, cerebral venous thrombosis
Thromboembolic disorders	Pulmonary embolism, deep vein thrombosis, superficial vein thrombosis
Extrapyramidal and movement disorders	Tremor, Parkinson-like disease, dystonia, myoclonus
Episodic disorders	Migraine, epilepsy and seizures, headache disorders
Neurologic-related disorders	Dizziness, somnolence, Guillain–Barré syndrome, encephalopathy, transverse myelitis
Peripheral nerve disorders	Peripheral neuropathy, paraesthesia, dysautonomia, Bell’s palsy
Sensory disorders	Hearing abnormalities or tinnitus, vision abnormalities
Cognition and memory	Alzheimer’s disease
Mental health disorders	Major depressive disorders, stress and adjustment disorders, anxiety disorders, psychotic disorders
Rheumatological disorders	Systemic lupus erythematosus, inflammatory myopathy
Other conditions	Diabetes mellitus, chronic kidney disease, chronic liver disease

*Source:* Please see the full reference list of the article Xie Y, Xu E, Bowe B, Al-Aly Z. Long-term cardiovascular outcomes of COVID-19. Nat Med. 2022;28(3):583–590. https://doi.org/10.1002/ski2.120, for more information

## Approach to the initial assessment

### Essential history-taking, clinical evaluation and functional assessment

The appropriate assessment of patients presenting with possible Long COVID is not yet fully outlined. While there are no evidence-based guidelines to direct the evaluation of these patients, a thorough history and clinical evaluation using a holistic patient-centred approach are required to characterise the nature of the complaints and to establish their possible relationship to prior SARS-CoV-2 infection. Documenting a history of confirmed or suspected SARS-CoV-2 infection is an increasingly important component of clinical practice and is central to recognising new or discrete presentations arising from a remote acute COVID-19 episode, especially when the patient has not volunteered such a connection themselves. Equally important is the systematic follow-up of patients with acute COVID-19 to ensure resolution of initial symptoms, recurrence of symptoms, or the development of new symptoms, especially within the 1st year following acute COVID-19. We recommend using standardised tools for this purpose, such as the WHO post-COVID-19 functional scale and the Montreal Cognitive Assessment (MOCA) test. In the absence of contemporary evidence-based local guidelines, clinical protocols should endeavour to include a comprehensive physical, cognitive and psychological assessment.^55,56,57,58^

### Who should be evaluated?

In patients with mild or moderate acute COVID-19 not requiring hospitalisation and who have demonstrated a progressive trajectory of clinical improvement during outpatient care, scheduled follow-up care is not necessary. However, patients should be encouraged to report new, persistent, worsening or recurrent symptoms, which should trigger a clinical consultation.^59^

Patients with mild to moderate COVID-19 who were self-managed in the community should be considered for a scheduled follow-up visit within 6 weeks of their acute illness.^59^

In patients who experienced severe acute COVID-19 requiring hospitalisation, scheduled follow-up care within 6 weeks of hospital discharge should be arranged to reduce the risk of rehospitalisation and to determine the recovery trajectory.^59^

In patients with new, persistent, worsening or recurrent symptoms, whether previously hospitalised or not, a dedicated Long COVID screening and assessment consultation is recommended.^59^

### When, where and how should patients be evaluated?

Initial Long COVID screening and assessment consultations should be scheduled approximately 12 weeks after acute infection, provided no life-threatening concerns are raised by the patient prior to this, and may be conducted in an outpatient primary care setting by a suitably qualified medical professional (nurse practitioner or general medical practitioner). Where available, these initial assessments should take place in dedicated multi-disciplinary clinics. Early specialist referral should be considered for patients who present with severe symptoms across multiple domains (such as physical, cognitive and mental health), those with atypical symptoms, or those who are experiencing severe functional impairment (social, educational or occupational).^59,60,61^

The consultation should aim to ascertain the following information: basis of the initial COVID-19 diagnosis, severity of the acute illness, pharmacological and non-pharmacological interventions used, nature of the initial symptoms, type and severity of complications (including venous thromboembolism, acute kidney injury, myocardial injury, superimposed sepsis, acute respiratory distress syndrome and delirium), and a detailed mapping of the trajectory of each of the symptoms between the onset of the symptoms and the Long COVID assessment. As far as is accurately possible, an assessment of the patient’s pre-COVID-19 clinical status should be established and used as a comparator. These assessments should be complemented by validated questionnaires for symptom characterisation, functional status, and social and occupational performance. The initial assessment should seek to exclude serious complications of acute COVID-19 and life-threatening conditions associated with Long COVID. These include myocarditis, myocardial ischaemia including microvascular angina, cardiac arrhythmias, interstitial lung disease, venous thromboembolic disease, cerebral venous thrombosis and hepatitis. A primary mental health diagnosis should not be made without review by a multi-disciplinary team or, at the minimum, a review by a neurologist. Furthermore, a suspicion of Long COVID should not supersede usual protocols and guidelines for the evaluation of undifferentiated symptoms, especially chest pain, dyspnoea, weakness and confusion. The identification of hypoxemia (oxygen saturation < 94%), oxygen desaturation on exertion (≥ 4%), unexplained tachycardia, hypotension, severe orthostatic hypotension, or altered mental status should prompt urgent referral to acute care services.^59,60,61^

Care should be taken to enquire about how a person’s life and activities have been affected by their symptoms, including their work or education, mobility and independence. An open, caring and empathetic acknowledgement of the impact of the illness on their day-to-day life is required.^59,60,61^

In patients with suspected Long COVID, initial bedside, laboratory and radiological investigations should be guided by the clinical evaluation, known comorbidities, abnormal results from the acute phase of the illness and existing symptom-based clinical algorithms.^59,60,61^

For patients who have fully recovered from acute COVID-19, no follow-up laboratory testing is needed. For individuals with an abnormal chest radiograph during the acute illness, follow-up chest imaging at 6 weeks is recommended for former and ongoing smokers.^59,60,61^

For patients with suspected Long COVID, it is reasonable to obtain a full blood count, kidney function test and electrolytes, liver function test including albumin, C-reactive protein, ferritin, thyroid function test and glycated haemoglobin.^55,56,57,58^ Further investigations should be directed by symptoms and clinical examination (Table 3). There is presently no evidence to support testing for SARS-CoV-2 infection by polymerase chain reaction assays, rapid antigen tests or antibody profiling.^59,60,61^

**TABLE 3:** Suggested investigations for common Long COVID phenotypes.

Investigation	Respiratory	Cardiac	Neurocognitive	Fatigue	Other
Full blood count	✔	✔	✔	✔	✔
Kidney function test	✔	✔	✔	✔	✔
Liver function test	✔	✔	✔	✔	✔
Thyroid function test	✔	✔	✔	✔	✔
Glycated haemoglobin	✔	✔	✔	✔	✔
C-reactive protein	✔	✔	✔	✔	✔
Ferritin	✔	✔	✔	✔	✔
WHO functional scale	✔	✔	✔	✔	✔
Wells score	✔	-	-	✔	-
D-Dimer	✔	-	-	✔	-
Spirometry	✔	-	-	✔	-
Chest radiograph	✔	✔	-	✔	-
Pulse oximetry	✔	✔	-	✔	✔
12-lead ECG	✔	✔	-	✔	✔
10-min standing test	-	✔	-	✔	-
Cardiac troponin	-	✔	-	✔	-
Brain natriuretic peptide	-	✔	-	✔	-
Validated cognitive assessment tool	-	-	✔	✔	-
Fatigue assessment scale	-	-	✔	✔	-
Sub-maximal exercise test	✔	✔	-	✔	-
Creatinine kinase	-	-	-	✔	-
Reactivated herpesvirus panel	-	-	-	✔	-
Morning cortisol	-	-	-	✔	-

ECG, electrocardiogram; WHO, World Health Organization.

## Focused clinical management

The mainstay of management is control of symptoms, appropriate intervention for treatable complications and detection of new medical diagnoses.^55,56,58^ For all patients with suspected Long COVID, it is important to optimise the pharmacotherapy for pre-existing or newly diagnosed comorbidities. The management of sleep disturbances, dermatological manifestations, gastrointestinal symptoms and mental health problems should follow the same protocols, practices and guidelines relevant in the absence of Long COVID.^1,59,60,62,63,64^

### Respiratory symptoms

For patients with persistent respiratory symptoms (including cough, dyspnoea, wheeze or pleuritic chest pain) at 12 weeks from the acute illness, it is reasonable to perform a chest radiograph, spirometry (if available at primary care level), and pulse oximetry at rest and following age-appropriate brief exercise. For patients with dyspnoea (especially subacute or acutely worsening dyspnoea), a validated tool for assessing the pre-test probability of pulmonary embolism (PE), such as the Wells Score, should be performed. In patients with a low pre-test probability of PE, performing a D-dimer test would help to exclude an event. Patients with a moderate or high pre-test probability of PE, or with a low pre-test probability of PE but an elevated D-dimer, should be referred to an acute care service for computed tomography pulmonary angiogram or ventilation-perfusion imaging.^1,59,60,62,63,64^

Patients with persistent radiographic abnormalities, hypoxemia at rest or desaturation on exertion should be referred to a pulmonologist for further evaluation. Patients with a normal initial assessment, but who remain symptomatic despite supportive measures at 6 months from the acute illness should also be referred to a pulmonologist for further evaluation. This might include evaluations of the diffusing capacity of the lung for carbon monoxide, whole body plethysmography, high resolution computed tomography of the chest, submaximal exercise testing and cardiopulmonary exercise testing. Dyspnoea, in particular, may warrant onward referral for cardiac evaluation.^1,59,60,62,63,64^ An isolated cough following recovery from acute COVID-19, after the exclusion of other common causes of a chronic cough (angiotensin-converting enzyme inhibitor use, gastro-oesophageal reflux disease and post-nasal drip), can be treated in the same way as other forms of post-viral tussis, using cough suppressants and/or inhaled bronchodilators.^1,59,60,62,63,64^ All patients with a chronic cough should also have tuberculosis excluded according to local diagnostic algorithms.

For patients who remain breathless in the absence of a treatable condition following specialist evaluation, structured pulmonary rehabilitation and breathing control techniques may be useful. The use of inhaled or oral corticosteroids for the treatment of persistent respiratory symptoms should only be considered as part of shared decision making with a pulmonologist and the patient.

### Cardiovascular symptoms

For patients with persistent cardiac symptoms (including anginal chest pain, atypical chest pain, dyspnoea, palpitations and presyncope) at 12 weeks from the acute illness, it is reasonable to perform a bedside 12-lead electrocardiogram (ECG), 10-min standing test and troponin. For patients with features of cardiac failure, N-terminal brain natriuretic peptide may be useful for supporting the diagnosis. Abnormal results should prompt a referral to a cardiologist for further evaluation. Patients with a normal initial evaluation, but who remain symptomatic despite supportive measures at 6 months from the acute illness should also be referred to a cardiologist for further evaluation. This might include an echocardiogram, exercise stress test, 24-h Holter monitoring, tilt-test, CT coronary angiogram, myocardial perfusion imaging, conventional coronary angiography and cardiac magnetic resonance imaging. Importantly, myocarditis cannot be ruled out on echocardiogram alone, and microvascular angina cannot be ruled out by either conventional coronary angiography or CT coronary angiogram.^1,59,60,62,63,64^

Persistent chest discomfort, in the setting of a normal pulmonary and cardiac evaluation, may benefit from treatment with a short course of non-steroidal anti-inflammatory drugs. For patients with predominantly orthostatic symptoms, especially those with a confirmed diagnosis of postural orthostatic tachycardia syndrome based on a 10-min standing test or tilt-test, conservative management with compression stockings, hydration and behavioural modification may be useful for symptom control. Pharmacological therapy with sodium-loading, heart rate limiting drugs, alpha receptor agonists or mineralocorticoids should only be considered as part of shared decision-making with a cardiologist and the patient.^1,59,60,62,63,64^

### Neurocognitive symptoms

For patients with persistent neurocognitive symptoms (including ‘brain fog’, memory impairment and difficulty concentrating) at 12 weeks from the acute illness, it is necessary to screen for cognitive impairment using a validated tool such as the MOCA test. Patients with severe or progressive impairment would benefit from a formal neuropsychological assessment. Brain imaging is not supported by the present evidence and should only be considered if the neurocognitive deficits are accompanied by focal neurological signs, concern for a space-occupying lesion, or as part of established evidence-based clinical algorithms for specific clinical presentations. Sleep quality and mental health should also be assessed in all patients reporting neurocognitive symptoms.

### Fatigue

Fatigue is universally the most common persistent symptom following acute COVID-19, and a substantial proportion of patients may ultimately satisfy the criteria for myalgic encephalomyelitis or chronic fatigue syndrome. This is also one of the most difficult symptoms for medical practitioners to investigate rationally, to provide supportive and symptomatic care for, to determine appropriate triggers for referral, and to determine who to refer the patient to. In patients who experience persistent fatigue or post-exertional malaise at 12 weeks from the acute illness, it is necessary to consider systemic contributors to the fatigue: sleep difficulties, endocrinopathies, metabolic disorders, nutritional deficiencies, autoimmune disease, cardiac disease, respiratory disease, anaemia and drug side effects (especially sedatives, hypnotics and antihistamines). The history, examination and initial investigations should be directed at excluding these potential contributors.^1,59,60,62,63,64^

Screening for fatigue can be performed using validated tools such as the Fatigue Assessment Scale (FAS) or Visual Analogue Fatigue Scale (VAFS). In addition, objective tests such as a sub-maximal exercise test (e.g., 6-min walk test) or the Timed Up-and-Go test may be useful for characterising the fatigue. Importantly, patients with post-exertional malaise may report worsening of the fatigue 12 h – 48 h and lasting for days or weeks after exertion, rather than at the time of the activity.^1,59,60,62,63,64^

In addition to the investigations outlined for the initial assessment and those described for respiratory and cardiac symptoms, it would be reasonable to request creatinine kinase, reactivated herpesvirus panels and morning cortisol. Abnormal results may help to identify those patients who require specialist referral for further diagnostic evaluation.^1,59,60,62,63,64^

All patients with persistent fatigue should be assessed against the diagnostic criteria for myalgic encephalomyelitis or chronic fatigue syndrome, and those meeting the criteria should be prioritised for management according to existing guidelines for this condition.^1,59,60,62,63,64^

The self-management of fatigue and post-exertional malaise should include rest, adequate sleep hygiene, energy conservation using specific evidence-based techniques. Structured rehabilitation programmes directed at a progressive and titrated return to activity may be suitable for most patients; however, a more individualised approach may be required for those who fail to advance through graded activity or those with severe post-exertional malaise.^1,59,60,62,63,64^

### Olfactory and gustatory symptoms

In patients with persistent anosmia or dysgeusia at 12 weeks from acute infection, it is reasonable to recommend olfactory training, using available online resources to guide self-management. Smoking cessation should be considered in ongoing smokers; iron deficiency should be excluded if the initial assessment reveals anaemia; and sinusitis and nasal polyps should be excluded as part of a complete ear, nose and throat examination. A nasal steroid spray may be considered as part of empiric supportive management. For patients with refractory symptoms, referral to an otorhinolaryngologist is recommended.^1,59,60,62,63,64^

## General principles

### Self-management and rehabilitation

The majority of patients with Long COVID benefit from either self-management or supported self-management. Support groups and patient organisations may be useful resources for reassurance, expectation-management and health information. Following a thorough clinical assessment, self-management should be recommended to suitable patients who do not require close clinical monitoring or upward referral. A recommendation of self-management should always be accompanied by advice about who to contact if symptoms become worrisome or if greater support is needed. Symptom diaries, including using mobile phone applications, should be encouraged.^60,64^

Multidisciplinary rehabilitation teams are best placed to construct individualised, patient-centred, rehabilitation plans, especially for those symptoms that are reported to persist into the long term (breathlessness, fatigue and brain fog). Ideally, these rehabilitation teams should foster shared decision making, supported self-management and deliver structured rehabilitation care with standardised monitoring, predefined outcome measurements, established referral plans and the option of home-based care. Patients with severe post-exertional malaise, exertional desaturation, orthostatic intolerance or cardiac impairment may not be suitable for physical exercise training. However, modified and individualised programmes can be considered by experienced teams.^60,64^

### Pharmacotherapy for the prevention or treatment of Long COVID

Two-dose vaccination reduces an individual’s risk of developing Long COVID by 34% compared to unvaccinated individuals.^65^ In addition, approximately 20% of patients who are vaccinated after the development of Long COVID report significant improvement in their symptoms and overall higher rates of resolution over time than unvaccinated individuals. As each reinfection with SARS-CoV-2 is associated with an increasing risk of Long COVID, in a dose-dependent fashion, the ongoing use of non-pharmacological measures to prevent infection should be encouraged.^66^

The use of nirmatrelvir-ritonavir within 5 days of symptom onset during acute COVID-19 reduces the risk of developing Long COVID by 26%, and represents another accessible, evidence-based intervention for the prevention of Long COVID.^67^ A breakthrough has been the finding that the use of metformin, started within 4 days of acute COVID-19 symptom onset and escalated to a dose of 1500 mg per day in divided doses over 2 weeks, reduces the risk of Long COVID by 40%, irrespective of disease severity, comorbidities and age.^68^ In discharged patients after hospitalisation for COVID-19 who are at high risk for venous thromboembolism, thromboprophylaxis with rivaroxaban 10 mg/day for 35 days has been shown to improve clinical outcomes.^69^

There are no registered drugs for the treatment of Long COVID after it has developed. However, several candidate agents are presently being investigated in over 30 registered clinical trials.

### Return to activity, exercise and work

The diverse effects of Long COVID may result in impaired stamina, standing, walking and cognition, which may translate into limitations in an individual’s ability to perform domestic, occupational, educational or social activities. Given the high degree of variability in the extent of impairment between patients with Long COVID and within Long COVID patients over time, it is reasonable to take an individualised approach to considerations related to return to independence in daily activities and return to work. Practitioners should be guided by formal occupational and functional assessments while formulating a strategy for returning a patient to work. A prolonged and flexible phased return to work may be necessary for some patients. Communication between medical practitioners, rehabilitation teams and employers should be encouraged and focused on establishing readiness for work, environmental modifications, the potential use of assistive devices and the production of a return-to-work action plan.

Care must be taken when assessing patients with persistent cardiorespiratory symptoms for return to work. In patients who experienced myocarditis during the acute illness or during the post-acute phase, especially high-performance athletes, a review by a cardiologist is necessary for guiding a safe return to activity.

## Conclusion

Long COVID is a complex medical condition with incompletely elucidated biological mechanisms underpinning its development, a diversity of clinical phenotypes, and significant intra-patient and inter-patient temporal heterogeneity. A rational, patient-centred, holistic approach that incorporates shared-decision making and multidisciplinary teams is necessary for the clinical care of patients with Long COVID.
